# Adverse Childhood Experiences in Children with Intellectual Disabilities: An Exploratory Case-File Study in Dutch Residential Care

**DOI:** 10.3390/ijerph15102136

**Published:** 2018-09-28

**Authors:** Jessica Vervoort-Schel, Gabriëlle Mercera, Inge Wissink, Emmelie Mink, Peer van der Helm, Ramón Lindauer, Xavier Moonen

**Affiliations:** 1 Koraal Center of Expertise, De Hondsberg, Hondsberg 5, 5062 JT Oisterwijk, The Netherlands; gmercera@koraalgroep.nl (G.M.); emmeliemink@gmail.com (E.M.); x.m.h.moonen@uva.nl (X.M.); 2 Department of Child Development and Education, University of Amsterdam, Nieuwe Achtergracht 127, 1018 WS Amsterdam, The Netherlands; I.B.Wissink@uva.nl; 3 Expert Center Social Work and applied Psychology, Professional University of Applied Sciences Leiden, Zernikedreef 11, 2333 CK Leiden, The Netherlands; helm.vd.p@hsleiden.nl; 4 Fier, National Expertise and Treatment Center, Holstmeerweg 1, 8936 AS Leeuwarden, The Netherlands; 5 Amsterdam UMC, University of Amsterdam, Department Child and Adolescent Psychiatry Meibergdreef 9, 1105 AZ Amsterdam, The Netherlands; r.lindauer@debascule.com; 6 De Bascule, Academic Center for Child and Adolescent Psychiatry, Meibergdreef 5, 1105 AZ Amsterdam, The Netherlands

**Keywords:** adverse childhood experiences, intellectual disabilities, children, behavior problems, youth psychopathology, physical health, family context, parents, residential youth care

## Abstract

Adverse Childhood Experiences (ACEs) are negative childhood events occurring in a child’s family or social environment, that may cause harm or distress. Children with intellectual disabilities (ID) and their families are underrepresented in international ACEs research, while current insights can also contribute to the improvement of their health and well-being. Deficiencies in intellectual and adaptive functioning and living circumstances can increase their vulnerability to adversities. In the present exploratory study 69 case-files of children referred to a Dutch national center for residential youth care for children with ID were analyzed to assess the prevalence and associations of ACEs. It was found that almost half (49.3%) of the children experienced 2 ACEs from the original ACEs framework or more (M (mean) = 2.1; SD (standard deviation) = 1.8) and that the number of ACEs in children was related to the presence of ACEs in parents. Both child and parental ACEs were also related to attachment- and trauma- and stressor-related disorders. Finally, living circumstances and multiple ACEs from the expanded ACEs framework, especially related to parental characteristics, were found to be related to ACEs in children with ID. This implicates the importance of a transgenerational approach when further investigating the impact of ACEs on mental and physical health in children with ID (intellectual disabilities).

## 1. Introduction

A global paradigm shift is currently taking place with regard to the understanding of health and disease throughout the human life span [1,2,3]. International research indicates that adverse childhood experiences (ACEs) play a key role in child development and subsequent adult health [4,5,6,7,8,9,10,11,12]. This new perspective on the foundations of health offers hopeful insights to promote health and mitigate negative health consequences [3,13], in which promising roles are reserved for relationships and emotion- and stress regulation, enhancing brain development and overall health [3,14,15,16]. Children with intellectual disabilities (ID) and their families are underrepresented in international ACEs research, while it is important that the current insights can also contribute to the improvement of their health and well-being [13,17,18,19].

Children with ID represent a heterogeneous group [20], differing in intellectual capacity, adaptive behavior and resilience, personality, abilities, environment, experiences and the (family) support they receive [21,22]. An intellectual disability is characterized by deficiencies in intellectual (IQ (intelligence quotient) < 70) and adaptive functioning, resulting in difficulties in reasoning, problem solving, abstract thinking and limitations in conceptual, social and practical adaptive skills [23,24,25,26]. Children with ID have been found to be more likely to be exposed to a wider range of adverse life events or traumatic events than their peers [17,19,27,28,29,30,31,32,33] as well. This exposure is also related to an increased risk of poorer mental health in individuals with ID [19,33,34,35,36,37,38,39,40,41,42,43,44].

The following sections will provide an introduction to ACEs and ID and the prevalence and impact on children’s health.

### 1.1. The ACEs Framework

Grounded on decades of prior research on early experiences and environmental influences [3], Felitti and colleagues in 1998 conclusively demonstrated the strong graded relationship between childhood adversity and mental and physical health in adulthood, using adult retrospective assessment [4,5,9]. In the first wave of their groundbreaking Adverse Childhood Experiences Study, experiences of abuse (physical, sexual, emotional) and household dysfunction (alcohol or substance abuse, mental health problems, domestic violence, parent incarcerated) were included [2,45]. Physical neglect, emotional neglect and parental divorce were added in the second wave of their study [2]. In general, ACEs originate from an accumulation of contextual factors and often cluster in children’s lives [6,8,45,46,47].

The studies on the original ACEs framework, demonstrating the profound long-term effects of unrecognized an untreated Adverse Childhood Experiences (ACEs) on health and well-being across the life span [11,15] have not only been replicated in the United States (U.S.), but also in many low-, middle- and high income countries all over the world [6,7,48]. Currently it is known that ACEs are associated with psychological and physical health problems, health risk behaviors and increased health care utilization, but also with developmental disruptions [49].

### 1.2. The Evolving ACEs Concept

Sources of ACEs originated initially from the family unit, but later researchers recommended expanding the ACEs framework to a wider range of adversities, including the surrounding environment: e.g., peer relationships [2,50,51,52,53,54], economic hardship [2,9,50,51,52,53,54,55,56], family relations [9,55], community stressors [2,9,51,52,53,54,55,57], negative school experiences [2,51], discrimination [2,50,52,53,54], separation from parents (e.g., hospitalization, foster care, institutional rearing) [9,51,52,53], and many (sudden) relocations [10,52,58].

The concept of ACEs is an overarching concept [28], and its development is a dynamic ongoing process, which already resulted in different proposed definitions [2,7,28]. In 2013 Kalmakis and Chandler clarified the ACEs concept for purposes of nursing research, theory development and practice, and proposed the following definition: “childhood events, varying in severity and often chronic, occurring in a child’s family or social environment that cause harm or distress, thereby disrupting the child’s physical or psychological health and development” [52] (p. 1489).

Examples of the further development of the ACEs framework are the recent hypotheses that also lower-level adversity like parental conflict [59] and spanking [60,61] can affect child development. Current research focuses on getting more consensus on the definition and the range of ACEs, aiming at improvement of international ACEs research [2,7,28]. Recently, Mclaughlin proposed the following working definition of childhood adversity: “exposure during childhood or adolescence to environmental circumstances that are likely to require significant psychological, social, or neurobiological adaptation by an average child and that represent a deviation from the expectable environment” [7] (p. 363). And despite a variety of ways and contexts in which the term resilience is used in the social, behavioral and biological sciences, the essence of resilience: “a positive, adaptive response in the face of significant adversity” [62] (p. 1), is part of the very latest proposed ACEs definition.

### 1.3. Prevalence of ACEs

Experiences of childhood adversity are found to be relatively common for children all over the world [9,63,64]. More than half of all children endure at least one type of adverse experience [64]. In European studies 14% to over 70% of the children and adolescents were reported to be exposed to at least one traumatic event [65]. In the Netherlands it was found that almost 50% of 10 to 11 year old children in regular primary schools experienced one or more adverse experience [66]. Exposure to 3–5 or more ACEs is considered to have strong associations with a broad range of severe health problems [6,67]. In the original ACE studies experiencing 4 ACEs was considered the threshold for extremely poor outcomes [68]. Children with ID are more likely to experience ACEs compared to children in the general population [28]. Research by Reichman and colleagues showed that having a disabling health condition, among which intellectual disability, was associated with 83% higher odds of the child experiencing 2 or more ACEs, and 73% higher odds of experiencing 3 or more ACE’s at age 5 [69]. Recent research indicates that the specific combination of exposure to poverty and parental mental illness may impact children’s health more than exposure to 3 or more ACEs [70].

### 1.4. The Vulnerability of the Child with ID

From a current social-ecological perspective, ID is viewed as a multidimensional state of human functioning in relation to environmental demands, involving the fit between capacities and context [24]. Adaptive functioning is a central theme in the recent definition of ACEs and resilience and is a key feature of intellectual disability. Few studies have investigated resilience in children with ID, but due to problems in executive functioning, self-regulation and problem solving, children with ID are less likely to be resilient than children in the general population [19]. For children and parents with intellectual disabilities, it can be complicated to successfully adapt to circumstances in their lives, increasing their vulnerability [23,24,71]. Once coping abilities of children and their parents are exceeded, severe problems can arise in the family, with detrimental effects on relations, development and health [8,46,72,73]. Individuals with an ID might be more susceptible to the disruptive consequences of life events and traumatization [32,41,44].

Given cognitive limitations and their vulnerability, differences may exist between individuals with ID and the general population in their experiences of adverse experiences [17]. There is evidence of a causal relationship between adverse life events and trauma symptoms in individuals with ID, however more research is necessary [43]. Martorell and Tsakanikos question how to differentiate between traumatic and life events; they suggest that maybe a clear cut-off point is not always possible [31]. They also question the role of adverse events: are these risk factors or triggering factors? [31]. More research is necessary considering the relationship between specific types and intensity of life events and traumatic events and the prevalence of mental health problems in individuals with ID [41,42].

Besides various cognitive and adaptive deficiencies, the living circumstances of children with ID can also have an impact on their vulnerability to adverse experiences. For example; as a result of developmental problems individuals with ID are at risk of social isolation [43], a high risk peer network [28] and vulnerability in social interactions [19,43]. Children with ID also have an increased likelihood of living in an institution, and remain more dependent on their caregivers throughout their lives [43,74]. Parental emotional reaction and parental support related to traumatic events are important mediators of the child’s health outcomes, especially in children with ID, given their dependency [74]. Lindblad and colleagues found that children born to mothers with ID were at high risk of adverse experiences and negative outcomes [75]. Individuals with ID were also found to be at risk for reduced access to support and healthcare [19,43]. Additionally, children with ID were found at risk of maltreatment as well [76], by family members or professional caretakers [28,77,78]. In fact, children with ID are presumably at least three times more likely to experience violence in their lives than their peers [28,78]. In families with children with ID, the risk of family violence is increased due to social, emotional and economic demands on the entire family [28]. The co-occurring behavioral problems in children with ID can result in increased parenting stress [79]. And possibly, the remaining dependency on caregivers makes the impact of interpersonal ACEs more severe [17].

### 1.5. The Influence of Parental ACEs

Recent studies examined the impact of parental ACEs on children in the general population. Overall, parental ACEs seem to have a transgenerational relationship with developmental problems in their children [80,81,82,83]. More parental ACEs and less resilience have been found to be associated with parental coping difficulties [84]. A study by Folger and colleagues showed that parental ACEs can negatively impact child development at 2 years of age on the following domains: communication, problem solving and personal- social- and motor skills [82]. Children of parents who experienced 4 ACEs or more, were 4 times as likely to have mental health problems [81]. Some studies have associated maternal life experiences and ACEs [81,85,86,87,88] and mental health [87] with the development and behavior of their children. Possibly, dysfunctional rearing behavior plays a mediating role in parental ACEs and children’s outcomes [80]. Impaired caregiving might play a role in the chronicity of the ACEs in children [68]. Parental ACEs may also influence attachment in the next generation, leading to difficulties in parenting and parent-child relationships [89,90]. Attachment was also found to have a mediating role in the associations between childhood adversity and health [91]. A study by Granqvist and colleagues showed that children of mothers with ID who experienced maltreatment, were significantly more likely to have high scores on disorganization and attachment insecurity measurements [92].

### 1.6. Impact on Children’s and Adolescents’ Health

From a developmental psychopathology perspective ACEs affect physical, emotional, behavioral, social and mental health and wellbeing [1,3,93,94,95,96], resulting in problems in attachment, behavior and emotion regulation [16,97], self-regulation, cognitive skills, language skills, social skills [8,64,94]. Research on these associations has emerged only recently [7]. The health threatening results of ACEs have not only been found in adult outcomes, but also in youth outcomes; like drug abuse [45,98,99], dissociation [45], reported higher rates of anger [45], anxiety [45], depression [45,98,99] and antisocial behavior [45,98], cancer, and liver disease [99]. Among young adolescents, physical abuse has been linked to health risk behavior, such as early pregnancy and smoking [100]. In a study by Peshevska et al. physical neglect increased the chances for drunk-driving, having more sexual partners, having early sex and for drug abuse [100]. As a result of ACEs, lower rates of engagement at school are also more likely [45,101] and there is an increased risk for learning- and behavioral issues and suicidal ideation [55]. Adolescence is a unique developmental stage, characterized by rapid growth in which physiologic, cognitive, social and emotional changes occur simultaneously, and ACEs may impede this development [55,102]. Finally, accumulation of ACEs can increase the risk of psychotropic medication in adolescents [103].

Below the age of 6, the most rapid brain development takes place and ACEs can have profound lifelong negative effects on this development [68,104]. A study among 3000 children in the U.S. revealed that exposure to ACEs was strongly associated with internalizing and externalizing behaviors and the likelihood of a diagnosis of ADHD (Attention Deficit Hyperactivity Disorder) in middle childhood [99]. This specific study revealed that children as young as 9 begin to show behavioral problems due to ACEs exposure [99]. A study by Kerker and colleagues revealed that in young children ACEs were associated with poor early childhood mental health and chronic medical conditions, and social developmental problems among children age 3–5 [105]. Research shows that persistent occurrence of ACEs in children has greater negative effects on internalizing and externalizing problem behaviors and on IQ than limited occurrences [45,106]. Childhood behavioral and emotional symptoms might be a crucial mediating link between ACEs and long term negative health outcomes [10,51]. Changes in brain structure and function [1,14,104,107] may be an underlying mechanism for these negative impact of ACEs, creating a weak foundation for later learning, behavioral problems and impaired health [1]. The neurodevelopmental trajectory of the association between ACEs and a range of negative health outcomes is still under investigation [108,109,110].

Focusing on psychopathology, worldwide nearby one-third of all mental disorders were considered as being attributable to ACEs exposure [7]. All classes of disorders at all life-course stages in all groups of WMH countries were found to be strongly associated with ACEs [111]. ACEs have demonstrated to increase the risk of depression, anxiety, PTSD (Posttraumatic Stress Disorder), aggression, suicide risk, personality disorders and behavior disorders [7,8,63]. In general, only after multiple traumas or with a history of anxiety, potentially traumatic events lead to PTSD symptoms in children [63]. 

In children with ID the risk of diagnostic overshadowing is present; attributing the emotional and behavioral problems to the disability instead of diagnosing a comorbid disorder [32,74], which can lead to underdiagnoses and inadequate interventions. The child’s intellectual functioning and language development are crucial in their reaction to traumatic experiences, like regressive behavior, aggressive and destructive behavior [74].

Despite existing research demonstrating the strong link between ACEs and various forms of (youth) psychopathology [7,8,64,95,112], greater understanding of the developmental pathways is necessary [7,12,45,109,113], especially for children with ID. Possibly, distinct dimensions of environmental experiences (e.g., deprivation, threat) influence development differently [7,12]. Mclaughlin et al. visualized this concept in a transdiagnostic model of childhood adversity and youth psychopathology [7].

### 1.7. The Present Study

In the present exploratory study, 69 case-files of children with intellectual disabilities referred to a national center for residential youth care in the Netherlands were analyzed. This research aims at making recommendations for further research in children with ID and their families. In the current study living circumstances from the sample were described and the associations among: (a) exposure to original adverse childhood experiences of children and their parents; (b) children’s problem behavior and youth psychopathology; (c) children’s physical health; and (d) other adversities and living circumstances related to the original ACEs framework and ID were explored. It was expected that the prevalence of ACEs in the sample group would be higher than the prevalence in the general population. The higher the number of original ACEs, the more physical health problems, problem behavior and youth psychopathology (especially aggression, anxiety and depression) were expected to be observed in the child. It was also expected that living circumstances as having a parent with an intellectual disability, ACEs or mental health problems, were associated with the presence of ACEs in children and with their health.

## 2. Materials and Methods

### 2.1. Sample

The study was approved by the Ethics Review Board of the University of Amsterdam (2018-CDE-8871). In this case-file study, data were analyzed of all 69 children with ID (46 male, 23 female) between the ages of 2 and 16 years old, who were discharged in 2016–2017 from “De Hondsberg”; a national center for residential youth care in the Netherlands for specialized clinical observation, diagnostics and treatment for children with intellectual disabilities and borderline intelligence and severe, persistent and complex mental- and behavioral health problems. The present study was part of a larger population study from De Hondsberg and only children with an IQ below 70 were included, conform the international definition of ID [25]. Before the data collection started, 3 children were excluded, due to the fact that they were discharged from the center before multidisciplinary research could have been finished, leading to incomplete case-files. Consequently, in the present sample it was made sure that all reports (see Section 2.2) needed for the data collection were present in the case-file. The present study was conducted between April 2018 and June 2018. Table 1 provides an overview of the socio-demographic- and other characteristics of the sample group. Multiple characteristics per individual are possible.

### 2.2. Procedure

Data were collected by two researchers of De Hondsberg from the digital archive using a generated structured case analysis system. Relevant variables were selected based on scientific literature on ACEs and on ID. Definitions, criteria and sources in the case files were well-defined in a codebook. The case-files included reports from previous and externally involved youth care settings as well as reports from the involved and individually adjusted multidisciplinary team of De Hondsberg, consisting of psychologists, psychiatrists, (system) therapists, pedagogues, physicians and residential care mentors. These reports included information about the child, parents and the family context the child grew up in. At De Hondsberg uniform standardized formats are used for reports of the multidisciplinary team. By excluding 3 case-files, as described in Section 2.1, it was made sure that all needed reports were present in the remaining case-files. The codebook was followed strictly as the presence of certain characteristics (for example an ACE or problem behavior) was only noted as present (’1’) in the data-file if the researchers found a description that matched the criteria as operationalized in the codebook. If the information about the variable did not match these criteria, the variable was scored as ’0’, meaning the information in the reports counteracted the presence of the characteristic in the child’s life. Data were collected in Excel and later transferred to SPSS. To calculate the inter-rater reliability, 28 case-files (20%) of the total case-file study were examined independently by both researchers. The inter-rater reliability was 97.2% which is considered high.

### 2.3. Measures

To explore the relationship between ACEs and mental- and physical health outcomes in children with ID and their parents, the ACEs from the original ACEs framework were used and operationalized, see Table 2. In the scientific literature several additional ACEs are suggested to expand the original ACEs framework (see Section 1.1 of the introduction), nonetheless we currently chose to focus our analysis mainly on the original ACEs framework because up until now, children with ID and their parents have been underrepresented in ACEs research. However, given the possible relevance of ACEs in the expanded ACEs framework, some additional ACEs (Number of placements in residential care, Problematic caregiver-child interaction, Hospitalization, Economic hardship, not attending school, Victim of bullying) were, based on the literature, included as independent variables to investigate their prevalence and relationship with physical and mental health outcomes. The additional ACE Economic hardship was defined as having at least 1 of the following living circumstances: Parents in debt, Housing problems or Unemployment of both father and mother. To determine behavioral problems and youth psychopathology in children, information was collected from the diagnostic reports of the multidisciplinary team of De Hondsberg, including: the outcomes of the Child Behavior Checklist 1.5-5/6-18 (CBCL) [114], the DSM-IV diagnosis [25], the presence of coping- and emotion regulation problems and the social-emotional level of functioning, assessed by the multidisciplinary team of De Hondsberg. Regarding the CBCL 1.5-5, only the syndrome scales corresponding to the syndrome scales in the CBCL 6-18 version were used. Multiple physical health variables were selected from the ACE literature and were included by analyzing the medical report from the physician at De Hondsberg. Other living circumstances, derived from scientific literature on ACEs and classifications based on the Dutch CAP-J [115], were included as possible risk factors for ACEs in children with ID (see Table 1).

### 2.4. Statistical Analyses

All statistical analysis were carried out using SPSS, version 23 (IBM, Armonk, NY, USA) Pearson correlations, independent samples *t*-tests and chi-square tests (for categorical variables) were used to assess the associations between different ACEs from the original ACEs framework, ACEs from the expanded ACEs framework, mental- and physical health outcomes and living circumstances. To explore the possible predictors of ACEs, a multivariate linear regression analysis was executed.

## 3. Results

### 3.1. Prevalence of ACEs from the Original ACEs Framework in Children with ID and their Parents

**Figure 1:**
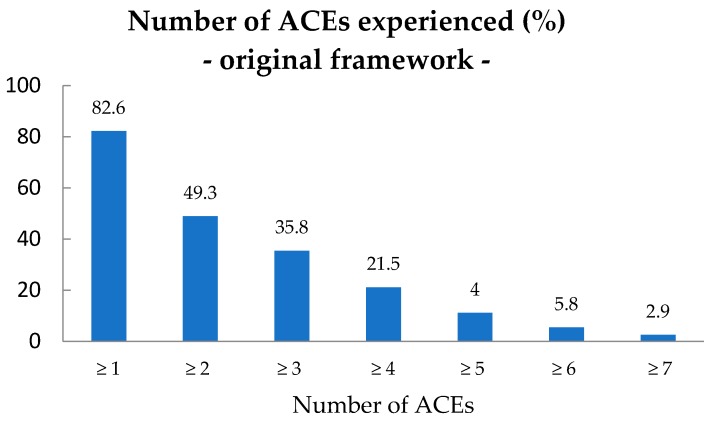
Number of ACEs from the original ACEs framework in children with ID, *n* = 69. ACEs: Adverse Childhood Experiences; ID: intellectual disabilities.

Of the children with ID, 57 (82.6%) had experienced at least 1 ACE from the original ACEs framework (M (mean) = 2.1; SD (standard deviation) = 1.8) as reported in their case-file. Figure 1 presents the prevalence (%) of the number of ACEs experienced from the original ACEs framework in children with ID. Most children (*n* = 23) experienced 1 ACE, however 34 children (49.3%) experienced 2 ACEs or more. The age (M = 11.3; SD = 3.1) of the children was normally distributed and no significant association was found between the number of ACEs and age (*p* < 0.05). Also, no significant association was found between the number of ACEs and gender of the child (*p* < 0.05).

**Figure 2:**
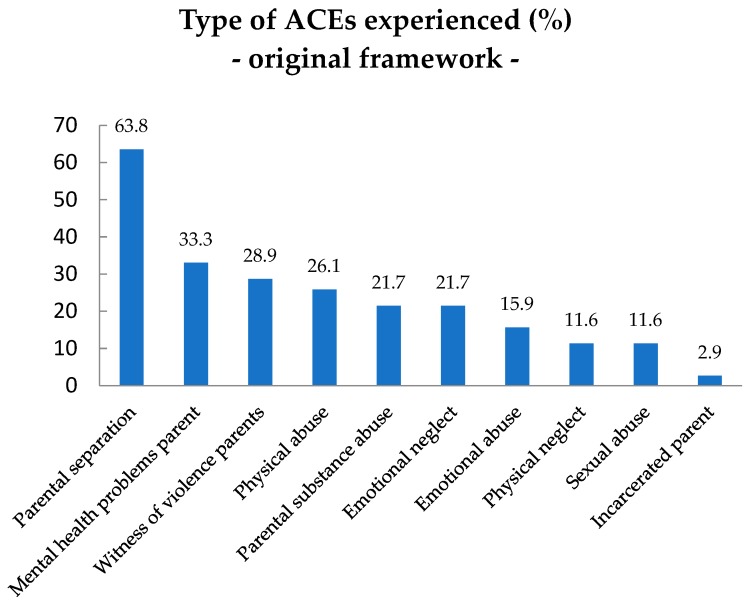
Type of ACEs experienced (%) from the original ACEs framework in children with ID, *n* = 69.

Figure 2 presents the prevalence (%) of type of ACEs from the original ACEs framework experienced by the children with ID. The most common ACEs were Parental separation/divorce (63.8%), followed by Parental mental health problems (33.3%) and Witness of violence against a parent (28.9%). The child having an Incarcerated parent was the least common original ACE to be experienced in the sample group (2.9%).

To explore the co-occurrence of ACEs from the original ACEs framework, Pearson correlation coefficients were calculated. As seen in Table 3, Emotional neglect appeared to be the most common ACE to occur together with other ACEs: most significant positive relationships were found between Emotional neglect and Emotional abuse, Physical neglect, Physical abuse, Parent’s substance abuse and Parent’s incarceration. Between Emotional neglect and Physical neglect a moderate relationship was found (0.577), whilst other significant relationships were weak.

A total of 20 children (28.6%) had Parents with ACEs. The presence of ACEs in parents correlated significantly with the Number of ACEs in children (0.317) and with the following specific ACEs: the presence of Parental substance abuse (0.360), an Incarcerated parent (0.270) and the child being a Witness of violence against parent (0.366). Using *t*-tests to further specify these findings, it was found that children of Parents with ACEs experienced more often Physical abuse (*p* = 0.045), Emotional neglect (*p* = 0.046), Parental substance abuse (*p* = 0.014) and they Witnessed more often violence against a parent (*p* = 0.007).

### 3.2. Associations between Original ACEs and Problem Behavior, Youth Psychopathology and Physical Health Problems

**Figure 3:**
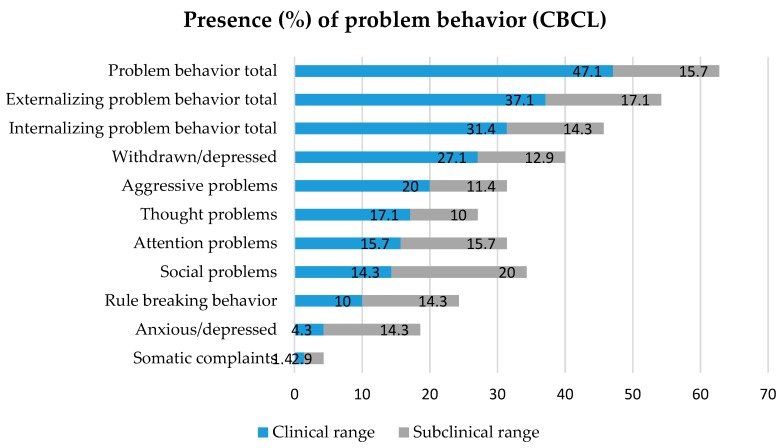
The presence of (sub)clinical problem behavior reported by residential care mentors in children with ID. CBCL: Child Behavior Checklist.

To explore problem behavior in children with ID, outcomes of the CBCL (Child Behavior Checklist) [114] questionnaire were used, as reported by the residential care mentors. The presence of clinical- and subclinical T-scores on the syndrome scales and broadband scales (Internalizing-, Externalizing- and Problem behavior total) were used as indicators for the occurrence of problem behavior (presence = 1, absence = 0). As seen in Figure 3, more than half (62.8%) of the children had a (sub)clinical score on the broadband scale of Problem behavior total. Focusing solely on clinical problem behavior, Externalizing problem behavior (37.1%) was slightly more common to be reported than Internalizing problem behavior (31.4%). Withdrawn/depressed problem behavior (27.1%) was the most present syndrome scale, followed by Aggressive problem behavior (20%). Anxious depressed problem behavior (4.3%) and Somatic complaints (1.4%) were least common in the sample group, however subclinical scores were relatively often reported on the Anxious/depressed syndrome scale.

Because of the relatively small sample size, not all assumptions for chi-square tests were met for the wide range of syndrome scales. Chi-square tests were therefore used in a explorative way to compare the clinical CBCL [114] outcomes to ACEs. This resulted in the following associations. Children whose Parent(s) had mental health problems, showed more Thought problems (*p* = 0.013) and children who were sexually abused showed more Rule-breaking behavior (*p* = 0.033) than children who did not experience these ACEs. More Somatic complaints were seen in children who experienced Physical neglect (*p* = 0.004), Emotional abuse (*p* = 0.014) and children with a (formerly) Incarcerated parent (*p* = 0.000). No significant differences (*p* < 0.05) were found in the Number of ACEs and the presence of problem behavior reported by the residential care mentors in the CBCL [114].

Independent-sample *t*-tests were used to compare the number of original ACEs to multiple DSM-IV outcomes related to aggressive behavior, anxiety, depression, attachment problems and trauma (DSM-5 was not yet used as classification resource in the years 2016–2017). A cut-off score of 2 for the dependent variable number of ACEs was used because of the mean (M = 2.1). Disorders were divided into the following categories: attachment-related problems and -disorders (33.3%), Trauma- and stressor-related disorders (32.9%), ADHD (21.4%), Disruptive-, impulse-control- and conduct disorders (2.9%), Mood disorders (1.4%) and Anxiety disorders (1.4%). Children who experienced 2 or more ACEs from the original ACEs framework, were significantly more often diagnosed with Attachment related problems and disorders (*p* = 0.000) and Trauma- and stressor-related disorders (*p* = 0.000) than children who experienced less than 2 ACEs. The mean difference was even significantly higher when a cut-off score of 4 ACEs was used, meaning that children with at least 4 ACEs from the original ACEs framework were more often diagnosed with Attachment and Trauma-related disorders than children with less than 4 ACEs. Between Attachment- related problems and disorders and Trauma- and stressor-related disorders a strong correlation was found (0.869). Also, ACEs in parents were related to more Attachment-related problems and disorders (*p* = 0.004) and Trauma- and stressor-related disorders (*p* = 0.004) in children who experienced 2 or more ACEs. No significant differences (*p* < 0.05) were found in the number of ACEs and the resulting DSM-IV categories and the total number of DSM-IV disorders.

Focusing on the characteristics of children with problem behavior it was also found that Sexual risk taking behavior was significantly related to the number of original ACEs in children. Using *t*-tests it was found that children with 2 or more ACEs from the original ACEs framework showed more often Sexual risk taking behavior (*p* = 0.047) than children with less than 2 original ACEs. No significant differences (*p* > 0.05) were found between the number of original ACEs and Suicidal ideation, Sexual rule-breaking behavior, the presence of Coping- and emotional regulation problems and the Social-emotional developmental delay.

Focusing on physical health characteristics of the child, Clinical hospitalization (34.3%) was most common, followed by Overweight or obese (30.4%) and Sleeping problems (27.5%). However, no significant differences (*p* < 0.05) were found between the Number of ACEs from the original ACEs framework and the physical health characteristics of the child (see also Table 1): Sleeping problems, Obstipation, Overweight or obese, Eczema, Headaches and/or stomach pains, Allergies and Respiratory symptoms.

### 3.3. Associations between ACEs from the Original and the Expanded ACEs Framework and Living Circumstances of the Child

**Figure 4:**
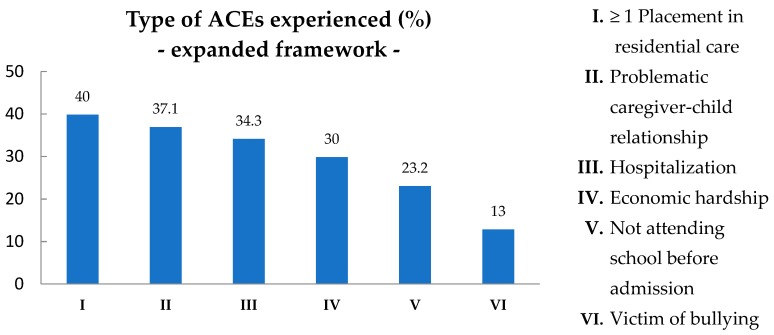
Type of ACEs experienced (%) from the expanded ACEs framework in children with ID, *n* = 69.

According to scientific literature on ACEs, ACEs from the expanded ACEs framework and living circumstances of the child are hypothesized to increase their vulnerability to adverse experiences (see introduction and Table 1). In Figure 4 the type of ACEs experienced (%) from the expanded ACEs framework are presented. 40% Of the children with ID had been placed in at least 1 residential care center or foster care home before admission to De Hondsberg. The mean Number of placements in residential care was 1.8. The added ACEs: Problematic caregiver-child relationship (37.1%), Hospitalization (34.3%) and Economic hardship (30%) were other common ACEs in the sample group.

Focusing on ACEs from the expanded ACEs framework using *t*-tests, it was found that children with 4 or more ACEs from the original ACEs framework, had a significant higher number of placements in residential care or foster care homes than children with less than 4 original ACEs (*p* = 0.015). Children with at least 3 original ACEs, had more often Parents in debt than children with less than 3 original ACEs (*p* = 0.001). Also it was found that children with at least 2 original ACEs had more often a Problematic relationship with their parent (*p* = 0.010) than children with less than 2 ACEs. No significant differences (*p* > 0.05) were found in the number of ACEs from the original ACEs framework and ACEs of the expanded ACEs framework: Hospitalization, Being bullied and Not attending school before admission. Other studies on ACEs often include ACEs from the expanded ACEs framework. When significant ACEs from the expanded ACEs framework were added to the ACEs from the original ACEs framework in the present study, the prevalence of at least two ACEs rose to 64.3% with a mean of 2.9 (SD = 2.2).

Focusing on the living circumstances of the child it was found that children with at least 4 ACEs from the original ACEs framework had more often Parents experiencing Limited parenting competence (as described by the professional in the case-file; *p* = 0.019) than children who experienced less than 4 original ACEs. Children with at least 3 original ACEs, had more often Parents who were involved with justice (incarceration excluded; *p* = 0.046) than children with less than 3 ACEs and children with 2 or more original ACEs had more often a Mother with ID (*p* = 0.024) than children with less original ACEs. No significant differences were found in number of original ACEs and the following living circumstances (*p* > 0.05): the Number of schools attended, Problematic caregiver burden and Having a limited social network.

As the present study found that both child characteristics and living circumstances of the child were related to the number of original ACEs, it was assessed how much of the variance in the number of original ACEs was explained by these different characteristics. A multivariate linear regression analysis was performed. Two groups of independent variables, selected based on their significance in the previous paragraphs, were divided into child- and living characteristics (Table 4). Model 2 contained the living characteristics and Model 3 contained the child characteristics. To control for Gender, Age and Country of birth, these variables were entered in Model 1.

Because of multicollinearity, the results were only interpreted on the model-level and not on the level of the variable coefficients independently. Results (the analyses were also performed using a Log transformation for the dependent variable because the assumption of normality was not met, however, the results of both analysis corresponded) showed that the total of variables in the models accounted for 72.8% of the variance in number of ACEs from the original ACEs framework. No significant influence of Gender, Age and Country of birth was found (*p* > 0.05). The living characteristics explained 38.2% of the variance in the number of ACEs from the original ACEs framework (*p* = 0.007). On top of that, child characteristics explained 30.5% of the variance in number of original ACEs (*p* = 0.001). According to these results, the differences in number of original ACEs in children with ID were explained by both child- and living circumstances.

## 4. Discussion

The present explorative case-file study was conducted to explore the prevalence of ACEs from the original ACEs framework in children with ID and their parents. The associations between the original ACEs, mental and physical health, ACEs from the expanded ACEs framework and living circumstances, were explored, aiming to make recommendations for further research in children with ID and their families. As children with ID and their families are underrepresented in international ACEs research, the results of the current study can fill gaps in the literature and improve the health and well-being of these children [13,17,18,19].

The case-file study revealed high prevalences of ACEs from the original ACEs framework in children with ID, as 82.6% of the children with ID experienced at least 1 original ACE. The mean number of ACEs from the original ACEs framework was 2.1 (SD = 1.8) and almost half (49.3%) of the children with ID experienced at least two original ACEs. In Europe 14% to 70% of the children and adolescents were reported to be exposed to at least one traumatic event [71] and in the Netherlands almost 50% of 10/11 year olds in regular primary schools were found to have experienced 1 or more adverse experiences [72]. Scientific literature on ACEs and ID describes that children with ID are more likely to experience 2 or 3 ACEs compared to children in the general population [28,69]. As expected for children with ID, the prevalences in the present study’s sample were higher than in the general population.

A considerable number of children had parents with ACEs (28.6%), which correlated positively with the total number of ACEs from the original ACEs framework in children and the presence of Parental substance abuse, Parental incarceration, the child being a Witness of violence against a parent and the child experiencing Physical abuse and Emotional neglect specifically. Because parental ACEs are assumed to have a transgenerational relationship with dysfunctional rearing behavior, parental coping problems and mental health problems in their children, including attachment problems [64,68,80,89,90], it is plausible that children of these parents are at higher risk for ACEs themselves.

Corresponding results were found focusing on youth psychopathology (DSM-IV diagnosis): children who experienced 4 or more ACEs from the original ACEs framework and children whose parents had ACEs, were more often diagnosed with Trauma- and stressor-related disorders and Attachment-related problems and disorders than children who had less than 4 original ACEs or no Parents with ACEs. The total prevalence of Trauma- and stressor-related disorders was 32.9%. Comparing this to the percentage of children in the sample size with 1 or more original ACE (82.6%) or the percentage of children with 2 or more original ACEs (49.3%), this number seems relatively low. According to scientific literature on ACEs and ID, trauma-related disorders are being missed frequently in individuals with ID as a result of diagnostic overshadowing [74], which means the professional attributes the problem behavior to a comorbid disorder leading to underdiagnoses. Also, children with ID often do not have the communication skills to identify and express their emotional states [74,116]. Therefore trauma symptoms might be unrecognized and are attributed to other challenging behavior, resulting in the individual not receiving the most optimal help.

The presence of problem behavior was, as expected giving their referral to De Hondsberg, common in the total sample, as 62.8% of the children scored on a (sub)clinical level on the CBCL Total problem behavior scale. It was found that children whose parents had mental health problems or children who experienced Sexual abuse, showed more Thought problem behavior and more Rule-breaking behavior respectively. Also Somatic complaints on the CBCL questionnaire were associated with the presence of Physical neglect, Emotional abuse and children with Incarcerated parents. However, these results should be interpreted carefully because assumptions for chi-square tests were not met due to the relatively small sample size.

It was expected that a wider range of ACEs were associated with the vulnerability of the child with ID for mental and physical health problems [2,9,10,50,51,52,53,54,55,56,57,58]. In the present study it seems that that multiple additional ACEs (Number of placements in residential care, Parents in debt and a Problematic caregiver-child relationship) and living circumstances of the child (a Mother with ID and Parents with ACEs, a Parent involved with justice and Parents experiencing limited parenting competence) were positively related to the presence of ACEs in children. As expected, the results showed a relationship between the presence of ACEs or ID in parents and the presence of ACEs in children and correspond to scientific literature on ACEs and ID, describing that children born to mothers with ID or parents with ACEs are at high risk of adverse experiences and attachment problems in which dysfunctional rearing and impaired caregiving play a mediating role [68,75,80,89,90,91,92]. Multivariate linear regression analysis underlined the importance of family living characteristics as both Child characteristics (30.5%) and Living characteristics (38.2%) explained a significant proportion in the number of ACEs.

According to scientific literature on ACEs, experiencing 3–5 or more ACEs is considered the threshold for strong associations with a broad range of severe (health) problems [6,67,68]. The present study found that this applied for the variables Attachment-related problems and disorders and Trauma- and stressor-related disorders, since the association was the strongest experiencing 4 ACEs. Also, children with at least 4 ACEs from the original ACEs framework had a higher number of placements in residential care, and children with at least 3 ACEs had the most often Parents in debt or a Parent involved with justice. Other associations between severe health problems and experiencing 3–5 ACEs were not found, as a threshold of 2 ACEs was most common for the presence of problematic behavior, ACEs from the expanded ACEs framework and living circumstances. A possible explanation is that the impact of ACEs in children with ID is more severe [32,41,44] and therefore less ACEs have the same health disrupting impact. A second explanation is that other studies on ACEs often include a wider range of ACEs from the expanded ACEs framework, causing a higher mean of the total number of ACEs experienced by the child. When adding the significant ACEs from the expanded ACEs framework to the ACEs from the original ACEs framework in the present study, the prevalence of at least two ACEs rose to 64.3% with a mean of 2.9 (SD = 2.2), so it is assumable that the mean number of ACEs will be higher when a wider range of adversities is applied.

Although the scientific literature on ACEs describes a connection between the presence of ACEs and problem behavior and youth psychopathology concerning aggression, anxiety and depression [45,47,98,99], the present study did not find such results. Yet Withdrawn/depressed behavior (40%) and Aggressive problem behavior (31.4%) were relatively often present in the outcomes of the CBCL questionnaire compared to other problem behavior. It is possible, besides the small sample size, that because of the overall complex behavioral problems of children with ID, it is hard to differentiate in problem behavior between children with different numbers of ACEs. Second, diagnostic overshadowing, might be an underlying cause of the relatively few diagnosed depressive-, anxiety- and aggressive related disorders. Also, because the results concern case-file information instead of (standardized) clinical interviews or screening tools, problems could be underrepresented or biased because of the great number of sources. At last, residential care mentors working with this specific group of children with ID for years might have developed a different frame of reference and may unknowingly compare the observed behavior to the most severe problem behavior among the children in the residential youth care center.

Unexpectedly, no significant associations were found between the Number of ACEs and medical characteristics of the child. As ACEs can have long-term effects on health and well-being across the life span [11,15], health outcomes possibly emerge at a later age. Also, in the final medical reports of De Hondsberg, the observations and results were summarized, so it is possible that not all present health problems were noted in these specific reports.

This study is not without limitations. As described earlier, the sample size was relatively small for the amount of characteristics which were investigated, therefore a risk of chance finding was present. Because of the small sample size, the number of optional statistical methods was limited, therefore no detailed analysis could be executed. Also the information has been collected from case-files instead of (standardized) clinical interviews or screening tools, causing underrepresentation or biased outcomes. Therefore follow-up research with a larger sample size and clinical interviews or screening tools, should further examine the association between ACEs and mental and physical health in groups of children with ID. Second, as De Hondsberg has a specialized and national function, only observing and treating the most severe cases of children with ID, other groups of children with ID should also be included in research. Also, the results of the present study suggest the need for an intergenerational approach [81,82,88,89,90] concerning ACEs in children, as both child characteristics and living characteristics possibly influence the number of ACEs experienced in children with ID. At last, because of their higher risk for ACEs and the fact that trauma related disorders are being missed frequently in individuals with ID, professionals in clinical youth care should be aware of the possible presence of ACEs and their profound impact on development and health. Early recognition can prevent ACEs and may contribute to mitigating their detrimental impacts. Standardized screening during intake might help identifying ACEs in children with ID and can contribute to providing the most optimal interventions to their problems.

## 5. Conclusions

The current study focused on exploring the association between ACEs and mental and physical health in children with ID and their parents, as they have been underrepresented in international ACEs research. It is important that this vulnerable group can also benefit from current international insights on health and disease. By means of an exploratory case-file search different insights have been gained to contribute to the improvement of the health and well-being of children with ID. The findings contain relevant theoretical and clinical implications to support the development of knowledge and insights for the field. The prevalence of ACEs from the original ACEs framework in children with ID was found to be relatively high comparing to the normal population. Also, ACEs from the expanded ACEs framework and specific living circumstances, were found to be related to the prevalence of original ACEs and attachment- and trauma related disorders. Acknowledging the negative associations between ACEs and the well-being, development and health of children with ID, can contribute to adequate assessment and choice of subsequent interventions to prevent and mitigate the long-term consequences of childhood adversity. This implicates the importance of a transgenerational approach in further research concerning ACEs in children with ID and their families. Further research is highly necessary and caution should be exercised in the use and interpretation of current results, as this was an exploratory study based on case-file information.

## Figures and Tables

**Table 1:** Socio-demographic- and other characteristics of the sample group (*n* = 69).

Variable	Percentage (*n*)
Child Characteristics	
Gender, % male	66.7% (46)
Age, mean (SD)	11.3 (SD = 3.1)
Nationality, % Dutch	92.8% (64)
Mean years of social-emotional developmental delay	6.3 (SD = 3.2)
Attachment problems (DSM-IV)	33.3% (23)
Trauma and stressor- related problems (DSM-IV)	32.9% (23)
Coping problems	17.4% (12)
Emotion regulation problems	62.3% (43)
Suicidal ideation	11.6% (8)
Sexual rule-breaking behavior	17.1% (12)
Sexual risk taking behavior	10% (7)
Average number of attended schools	2.9 (SD = 1.4)
Physical health characteristics of the child	
Experienced clinical hospitalization	34.3% (24)
Sleeping problems	27.5% (19)
Obstipation	13% (9)
Overweight or obese	30.4% (21)
Eczema	15.5% (10)
Headaches and/or stomach pains	12.9% (9)
Allergies	7.1% (5)
Respiratory symptoms (asthma, bronchitis)	5.7% (4)
Use of psychotropic medication	55.7% (39)
Sleep medication and tranquillizers	32.9% (23)
Antipsychotic medications	30% (21)
Stimulants	18.6% (13)
Anti-depressants	4.3% (3)
Family characteristics	
Biological parent(s) with ACEs	28.6% (20)
Biological parent(s) with an ID	27.1% (19)
Physical health problems parent(s) *	31.9% (22)
Parent(s) involved with justice (incarceration excluded)	8.6% (6)
Divorced or separated biological parents	63.8% (44)
New composed family	37.7% (26)
One parent household	34.8% (24)
Average number of primary caregivers	2.4 (SD = 0.8)
Average number of (step)siblings grown up with	2.8 (SD = 1.4)
Child protection/welfare involved	33.3% (23)
Parent(s) in debt	23.2% (16)
Parent(s) with housing problems	7.2% (5)
Unemployment father	15.9% (11)
Unemployment mother	36.2% (25)
Limited social network	30% (21)
Problematic caregiver burden	70% (49)
Limited parenting competence	24.3% (17)
Problematic caregiver-child relationship	37.1% (26)
Residence before admission to De Hondsberg	
Living with parent(s)	66.6% (46)
Residential youth care	14.3% (10)
Crisis intervention residence	11.6% (8)
Foster care	4.3% (3)
Family	1.4% (1)

* note: the term parent refers to the primary caregiver (not necessarily the biological parent).

**Table 2:** Overview and definition of the original ACEs (Adverse Childhood Experiences) framework used in the present study.

ACEs	Definition
Physical abuse	Being pushed, beaten, grabbed, slapped, kicked or being hit so hard resulting in marks or injury
Emotional abuse	Being sworn at, insulted, threatened, put down
Physical neglect	Parent’s or primary caregiver’s behavior interfered with the child’s care, wearing dirty clothes, bad hygiene, not enough personal living space, no safe living space, not enough to eat, not taken to a doctor when sick, forced to take care for themselves
Emotional neglect	Parents didn’t make the child feel special and loved, the family not being a source of strength, protection and support, the child receiving little attention
Sexual abuse	Being involuntarily touched in a sexual way, forced into any form of sexual contact, forced into watching sexual content
Parental incarceration	A parent or primary caregiver being incarcerated
Parental separation/divorce	Separation or divorce of biological parents
Witness of violence against a parent	The child being a witness of verbal or physical violence (abuse) against the parent or primary caregiver
Parental substance abuse	Excessive alcohol use or drug use of the parent or primary caregiver
Parental mental health problems	Biological parent(s) having mental health problems (anxiety, depression, bipolar disorder or other mental issues/illnesses) interfering with the child’s care or having a parent ever attempted suicide

**Table 3:** Correlation coefficients of the dichotomous original ACEs framework variables.

ACEs Variables	1	2	3	4	5	6	7	8	9	10
1. Emotional neglect M = 0.22; SD = 0.415	1	-	-	-	-	-	-	-	-	-
2. Emotional abuse M = 0.16; SD = 0.6	0.346 **	1	-	-	-	-	-	-	-	-
3. Physical neglect M = 0.12; SD = 0.323	0.577 **	0.213	1	-	-	-	-	-	-	-
4. Physical abuse M = 0.26; SD = 0.442	0.327 **	0.463 **	0.300 *	1	-	-	-	-	-	-
5. Sexual abuse M = 0.12; SD = 0.32	0.136	0.211	0.008	−0.012	1	-	-	-	-	-
6. Substance abuse M = 0.22; SD = 0.41	0.319 **	0.154	0.138	0.087	0.266 *	1	-	-	-	-
7. Mental health pr. M = 0.33; SD = 0.47	0.075	0.112	0.032	0.070	0.040	0.298 *	1	-	-	-
8. Witness violence M = 0.2; SD = 0.46	0.059	0.213	0.145	0.435 **	−0.150	0.199	−0.054	1	-	-
9. Incarceration M = 0.03; SD = 0.169	0.328 **	0.397 **	0.477 **	0.094	0.207	0.118	0.244 *	0.126	1	-
10. Divorce M = 0.64 SD = 0.484	0.251 *	0.164	0.179	0.242 *	−0.006	0.178	0.021	0.049	−0.049	1

** correlation significant at the 0.01 level. * correlation significant at the 0.05 level.

**Table 4:** Significant living circumstances and child characteristics (< 0.05) on the number of ACEs in children with ID.

Significant Living Characteristics	Significant Child Characteristics
Parents in debt	Attachment related problems/disorders
ACEs in parents	Trauma- and stressor- related disorders
Mother with ID	Number of placements in residential care or foster care homes
Parent experiencing limited parenting competence	Sexual risk taking behavior
A parent in contact with justice (incarceration	Rule-breaking behavior (CBCL)
excluded)	Thought problems (CBCL)
Problematic caregiver-child relationship	Somatic complaints (CBCL)
